# The effect of blood flow restriction training combined with electrical muscle stimulation on neuromuscular adaptation: a randomized controlled trial

**DOI:** 10.3389/fphys.2023.1182249

**Published:** 2023-05-05

**Authors:** Na Li, Jingfeng Yang, Yuanpeng Liao

**Affiliations:** ^1^ National Clinical Research Center for Geriatrics Diseases, West China Hospital, Sichuan University, Chengdu, Sichuan, China; ^2^ Department of Sports Medicine and Health, Chengdu Sport University, Chengdu, Sichuan, China

**Keywords:** blood flow restriction, electromyostimulation, muscle activation, muscle strength, muscle hypertrophy

## Abstract

**Objective:** Low-intensity resistance training (≤25% 1RM) combined with blood flow restriction training (BFRT) is beneficial to increasing muscle mass and muscle strength, but it cannot produce increased muscle activation and neuromuscular adaptation, as traditional high-intensity strength training does. The purpose of this study is to investigate the effects of independently applying BFRT and electrical muscle stimulation (EMS), as well as combining the two methods, on muscle function.

**Methods:** Forty healthy participants with irregular exercise experiences were randomly assigned to four groups: BFRT-alone group (BFRT, *n* = 10), EMS-alone group (EMS, *n* = 10), BFRT combined with EMS group (CMB, *n* = 10), and the control group (CTR, *n* = 10). All participants received low-intensity squat training at a load of 25% 1RM 5 times/week for 6 weeks. Cross-sectional area (CSA) and electromyographic root mean square (RMS) in the rectus femoris, as well as peak torque (PT) of the knee extensor, were measured before and following a 6-week intervention.

**Results:** Following the 6-week intervention, the increases in muscle activation in the CMB group were statistically higher than those in the BFRT group (*p* < 0.001), but not different from those in the EMS group (*p* = 0.986).

**Conclusion:** These data suggest that the combination of BFRT and EMS for low-intensity squat training improved the muscle strength of the lower limbs by promoting muscle hypertrophy and improving muscle activation, likely because such a combination compensates for the limitations and deficiencies of the two intervention methods when applied alone.

## 1 Introduction

Loss of muscle mass in the lower extremities and reduced muscle strength due to decreased neuromuscular adaptation are important causes of serious health problems such as falls, disability, and even death (Visser et al., 2005). Squat training is an effective method to prevent muscle atrophy and improve muscle strength in the lower extremities (Kraemer et al., 2017). According to the American College of Sports Medicine (ACSM), resistance strength training at a load of ≥70% 1-repetition maximum (1RM) can effectively induce muscle hypertrophy and improve muscle strength (Kraemer et al., 2009). However, for postoperative rehabilitation patients, athletes with skeletal muscle injuries, the elderly, and other populations with special needs, high-load, and high-intensity resistance training is not practical. In addition, for ordinary people who have no prior resistance training experience, such training may increase the risk of sports injury (Gardiner et al., 2020).

Blood flow restriction training (BFRT) is a method of applying equipment with special material to exert pressure on the proximal end of the limbs during exercise to enhance the training effect. Numerous studies have confirmed that low-intensity resistance training (≤25% 1RM) combined with BFRT is beneficial for increasing muscle mass and muscle strength (Colomer-Poveda et al., 2017; Korakakis et al., 2018; Ladlow et al., 2018). Therefore, BFRT can be used as a recommended method for people who are unable to perform traditional high-load resistance strength training. However, BFRT has an inherent limitation. The underlying mechanism of BFRT-based strength training is dominated by muscle hypertrophy, as it cannot produce increased muscle activation and neuromuscular adaptation, as traditional high-intensity strength training does (Manini and Clark, 2009; Cook et al., 2013).

Electrical muscle stimulation (EMS) uses a low-frequency rectangular pulse electric current to stimulate a specific muscle, resulting in involuntary muscular contraction. Numerous studies have shown that EMS is beneficial for improving muscle strength (Šarabon et al., 2020; Kemmler et al., 2021; Nishikawa et al., 2021). However, there is no consensus on the existence of an “optimal” frequency range. Filipovic et al. stated that frequencies of approximately 76 Hz lead to optimal strength development (Filipovic et al., 2011). Previous studies suggested that the muscle strengthening seen following EMS results from a reversal of voluntary recruitment order with a selective augmentation of type-II muscle fibers (Gregory and Bickel, 2005; Jubeau et al., 2007; Sheffler and Chae, 2007). Because type-II fibers have a higher specific force than type-I fibers, selective augmentation of type-II muscle fibers will increase the overall strength of the muscle. However, the main limitation of EMS for inducing muscle hypertrophy and improving muscle strength is directly related to the stimulation intensity. The intensity required to effectively increase strength and hypertrophy is particularly prone to induce prohibitive pain and rapid muscle fatigue (Filipovic et al., 2011), thus reducing the participants’ compliance. Recent studies found that the combination of BFRT and low-intensity EMS might avoid the discomfort caused by high current intensity when EMS has been applied alone (Natsume et al., 2015; Slysz and Burr, 2018; Slysz et al., 2021).

Therefore, the purpose of this study was to investigate the effects of BFRT alone, EMS alone, and BFRT + EMS on muscle volume, muscle activation, and muscle strength. Low-intensity squat training (≤25% 1RM) was used as a control. By comparing the changes in the parameters related to lower limb muscle volume, muscle activation, and muscle strength of subjects under different intervention methods, the effects of each intervention method were confirmed and the related mechanisms of their influence on muscles were clarified, to provide a research basis for the promotion and application of combined squat training in clinical rehabilitation. We hypothesize that the combination of BFRT and EMS will lead to a greater training effect than either of these interventions in isolation.

## 2 Materials and methods

### 2.1 Participants

A total of forty healthy young men were recruited from the Chengdu Sport University. The inclusion criteria were as follows: 1) males, 2) good physical health (screening exercise risk through the PAR-Q), 3) low levels of physical activity (scored by the IPAQ), 4) naive to squat training, BFRT training, or EMS training and 5) absence of lower limb injury. After being advised of the purpose and potential risks of the study, all participants provided written informed consent, and the study was approved by the ethics subcommittee of Chengdu Sport University (CDSUEC_2020_17_57) and conducted in accordance with the Declaration of Helsinki.

### 2.2 Study design

This was a randomized study. Random sequences generated by the SPSS version (SPSS Inc., United States) were hidden in consecutively numbered (1–40), sealed, opaque envelopes, where the allocation ratio was 1:1:1:1. Participants were randomly allocated to either the BFRT-alone group (BFRT, *n* = 10), the EMS-alone group (EMS, *n* = 10), the BFRT combined with EMS group (CMB, *n* = 10), or the control group (CTR, *n* = 10). When the researchers confirmed that the participants met the inclusion/exclusion criteria, the envelopes were opened in sequence and the participants were assigned to the corresponding groups. All outcomes were measured by rehabilitation therapists with standardized training and professional technical qualifications, and each participant was measured by the same evaluator before and after the intervention to avoid errors.

### 2.3 Intervention

**FIGURE 1:**
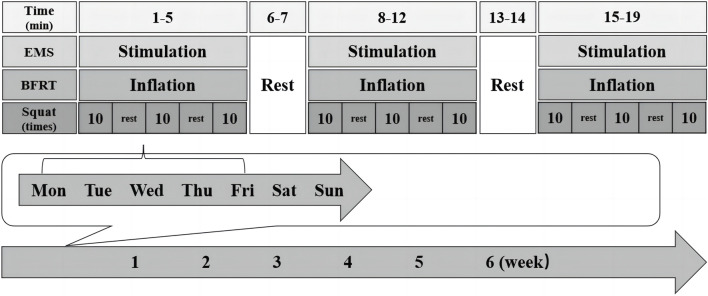
The timeline of the entire experimental intervention. All participants underwent exercise intervention for 19 min/session, once/day, 5 days/week, and lasted for 6 weeks.

Each participant underwent an intervention protocol consisting of 30 19-min sessions, which took place once per day, 5 days a week, and lasted for 6 weeks (Figure 1). Each session was supervised by the same researcher. All experiments were carried out at the Institute of Sports Medicine and Health of Chengdu Sport University.

#### 2.3.1 CTR group

High-bar squat training (Murawa et al., 2020) at 25% 1RM was performed in the first, third, and fifth minutes of the session, and the rest was performed in the second and fourth minutes. The rhythm of the squat was controlled by a metronome 10 times/min (3 s concentric/3 s eccentric). Squat 30 reps per set, with a 2-min interval, and repeat for 3 sets. At the end of the second and fourth weeks of intervention, 1RM was reassessed periodically to appropriately adjust for resistance.

#### 2.3.2 BFRT group

**FIGURE 2:**
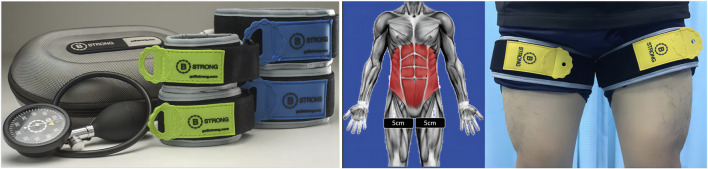
The BFRT group. A 5-cm wide tourniquet cuff was placed at the groin crease, and according to the leg circumference inflated to the appropriate pressure (≤50 cm, 200 mmHg; 51–55 cm, 250 mmHg; 56–59 cm, 300 mmHg; ≥60 cm, 350 mmHg). Each session comprised 3 cycles of 5 min of inflating and 2 min of deflating.

BFRT was performed with an automated tourniquet system (B-strong, United States). The squat program was the same as that of the CTR group. Meanwhile, a 5-cm wide tourniquet cuff was placed at the groin crease, and according to the leg circumference inflated to the appropriate pressure (≤50 cm, 200 mmHg; 51–55 cm, 250 mmHg; 56–59 cm, 300 mmHg; ≥60 cm, 350 mmHg) (Jia Wei et al., 2019). Each session comprised 3 cycles of 5 min of inflating and 2 min of deflating. As shown in Figure 2.

#### 2.3.3 EMS group

**FIGURE 3:**
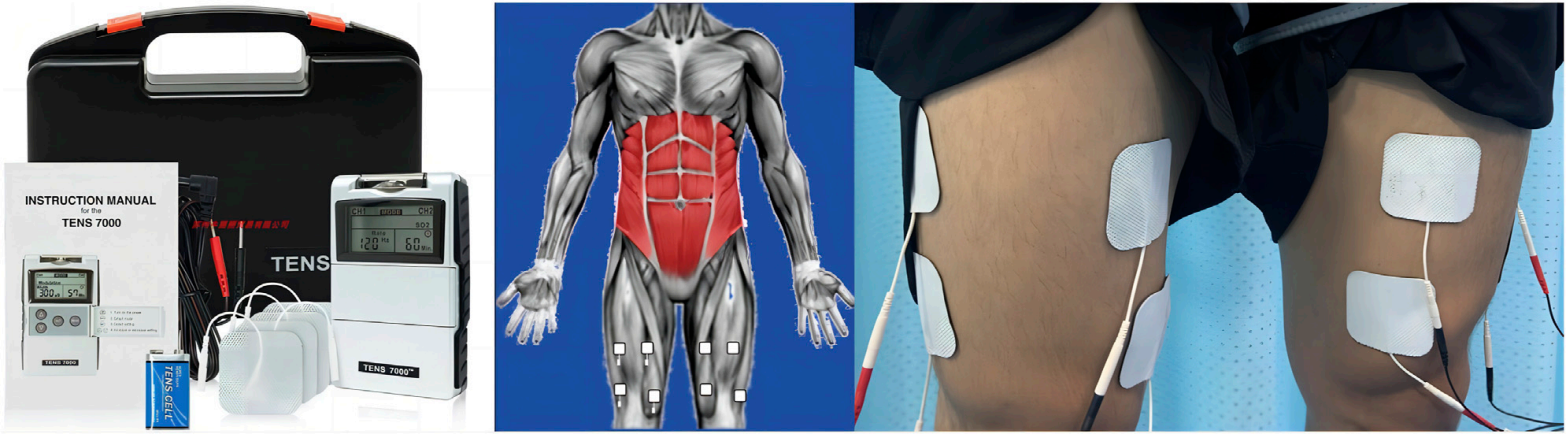
The EMS group. Two negative electrodes (5 × 5 cm) were placed near the groin crease approximately 10 cm below, and the other two positive electrodes (5 × 5 cm) were placed as close as possible to the movement point of the vastus lateralis and medial muscles. The EMS protocol consisted of a duty cycle of 3 s ON (stimulation) and 3 s OFF (no stimulation) with the pulse width set at 400 µs, the intensity set at 50 mA, and the stimulation frequency set at 75 Hz. Each session comprised 3 cycles of 5 min of stimulation and 2 min of rest.

EMS was performed with an electrical muscle stimulation instrument (Twin Stim Plus: Tens Unit 7,000, United States). The squat program was the same as that of the CTR group. The EMS protocol consisted of a duty cycle of 3 s ON (stimulation) and 3 s OFF (no stimulation) with the pulse width set at 400 µs, the intensity set at 50 mA, and the stimulation frequency set at 75 Hz (Filipovic et al., 2011; Seyri and Maffiuletti, 2011). Before performing the squat, an exercise pointer was used to accurately locate the most obvious point of muscle contraction. Then, two negative electrodes (5 × 5 cm) were placed near the groin crease approximately 10 cm below, and the other two positive electrodes (5 × 5 cm) were placed as close as possible to the movement point of the vastus lateralis and medial muscles. Each session comprised 3 cycles of 5 min of stimulation and 2 min of rest. As shown in Figure 3.

#### 2.3.4 CMB group

**FIGURE 4:**
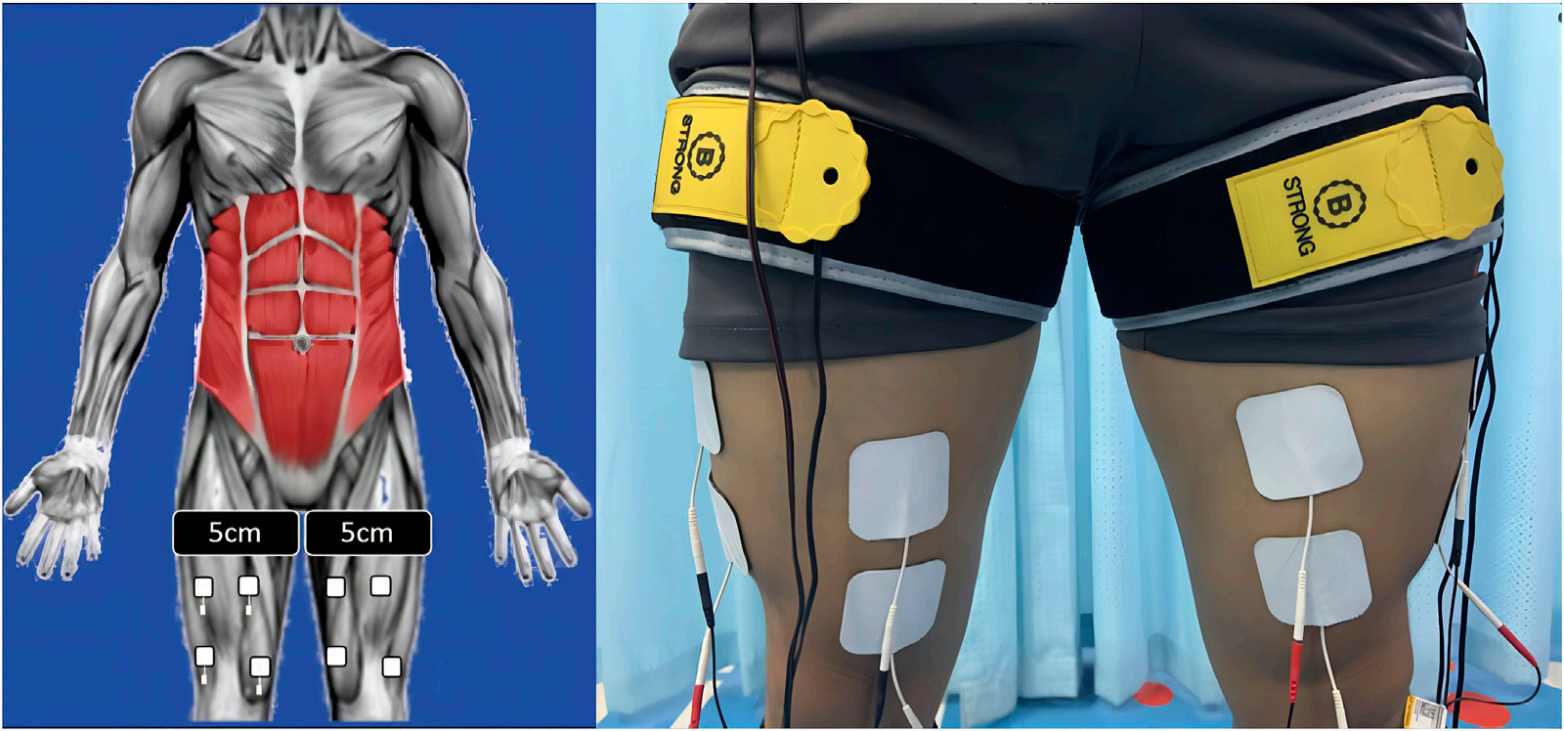
The CMB group. In the CMB group, BFRT and EMS were carried out simultaneously in the same way as above. The squat program was the same as that of the CTR group.

BFRT and EMS were carried out simultaneously in the same way as above. The squat program was the same as that of the CTR group. As shown in Figure 4.

### 2.4 Outcomes

#### 2.4.1 Muscle strength and muscle activation

The muscle activation test was performed at the same time as the isokinetic muscle strength test. Before the test, participants underwent a 10-min warm-up session. A wireless surface electromyography (sEMG) system (Myon 320, Schwarzenberg, Switzerland) was used to collect electrical signals from muscles during exercise. Electrodes (Ag/AgCl) were affixed to the rectus femoris on both sides of the participant, and the electrodes were 2-3 cm apart. Then, the participants were asked to sit relaxed, and the baseline signals and signal quality of all electrode signals were detected through knee flexion and extension. Then, all participants underwent strength testing of the knee extensors using an isokinetic dynamometer (Con-Trex MJ, Switzerland). Knee extension maximal voluntary contractions were measured at 90° with participants’ seats at 90° hip flexion. Each leg was positioned on the dynamometer by aligning the participants’ knee with the axis of the lever arm and attaching the dynamometer arm approximately 3 cm proximal to the medial malleolus. Participants were instructed to contract as “fast and forcefully as possible”. Standard verbal encouragement and visual feedback were provided during each contraction. Before the formal test, participants tried 3 moderate strength exercises to familiarize themselves with the procedure. The test speed was set at 30°/s, which was repeated for 5 contractions.

The outcomes of muscle strength were selected as the peak torque (PT) of knee extension. The sEMG signal was analyzed in the time domain, and the selected index was the root mean square (RMS). sEMG preprocessing: The raw sEMG sampling frequency was 2000 Hz, and the sEMG data were preprocessed by low-pass and high-pass filtering (frequency 10–400 Hz) and full-wave rectification. To avoid the influence of different participants’ RMS before and after the experiment, such as the position of electrode paste and cortical thickness, the maximum normalization method was used to standardize the RMS values.

#### 2.4.2 Muscle thickness

The cross-sectional area (CSA) of the rectus femoris was assessed via ultrasound (PHILIPS, Netherlands) as an index of the whole rectus femoris muscle volume. The measurement position was supine. The site was landmarked to ensure consistency of measures (from the midpoint of the patella to the middle of the 30%, 50%, and 70% cross-sections of the greater trochanter of the femur). The average value of the three locations was taken as the final result. Using B-mode ultrasound with a linear-array probe (L12-3, 3–12 MHz, Philips) with minimal pressure was applied to avoid compression of the underlying tissue. Images were analyzed immediately post-acquisition, and muscle thickness was assessed by measuring the distance (cm) from the superficial to the deep aponeurosis at the exact center of the fasciae (QLAB Software, PHILIPS). The final CSA of the rectus femoris is the average of the left and right sides.

### 2.5 Statistical analysis

The descriptive statistics are reported as the mean ± standard deviation ( $\bar{x}$ ± SD ). The difference (Delta) between the pre- and post-intervention muscle volume, muscle activation, and muscle strength were calculated between week 0 and week 6. For each outcome measure, a one-way ANOVA was performed. The dependent variable was the Delta of the specific outcome measure, and the factor was the Intervention Methods, which had four levels: CTR, BFRT, EMS, and CMB. In case of significant *F*, a Bonferroni *post hoc* analysis will be performed to assess the difference across the four intervention methods. Statistical significance was set at *p* < 0.05, and all analyses were performed with SPSS version 25.0 (SPSS Inc., United States).

## 3 Results

### 3.1 Descriptive characteristics

The descriptive characteristics of 40 participants who completed the entirety of the 6-week intervention are provided in Table 1. Before the intervention, no significant differences were observed between the four groups in terms of age (*p* = 0.736), height (*p* = 0.589), weight (*p* = 0.604), and body mass index (BMI, *p* = 0.490).

**TABLE 1:** Descriptive characteristics of the participants.

Items	CTR	EMS	BFRT	CMB	*p*-value
	(*n* = 10)	(*n* = 10)	(*n* = 10)	(*n* = 10)	
Age (years)	20.9 ± 1.1	20.8 ± 1.5	20.4 ± 1.7	20.5 ± 1.0	0.736
Height (cm)	172.4 ± 8.1	171.6 ± 6.9	172.7 ± 6.4	170.6 ± 9.4	0.589
Weight (kg)	67.6 ± 10.2	67.2 ± 9.7	65.3 ± 11.2	66.1 ± 9.1	0.604
BMI (kg/m^2^)	20.9 ± 2.1	21.3 ± 2.4	21.1 ± 2.7	20.7 ± 3.0	0.490

### 3.2 Muscle volume

**FIGURE 5:**
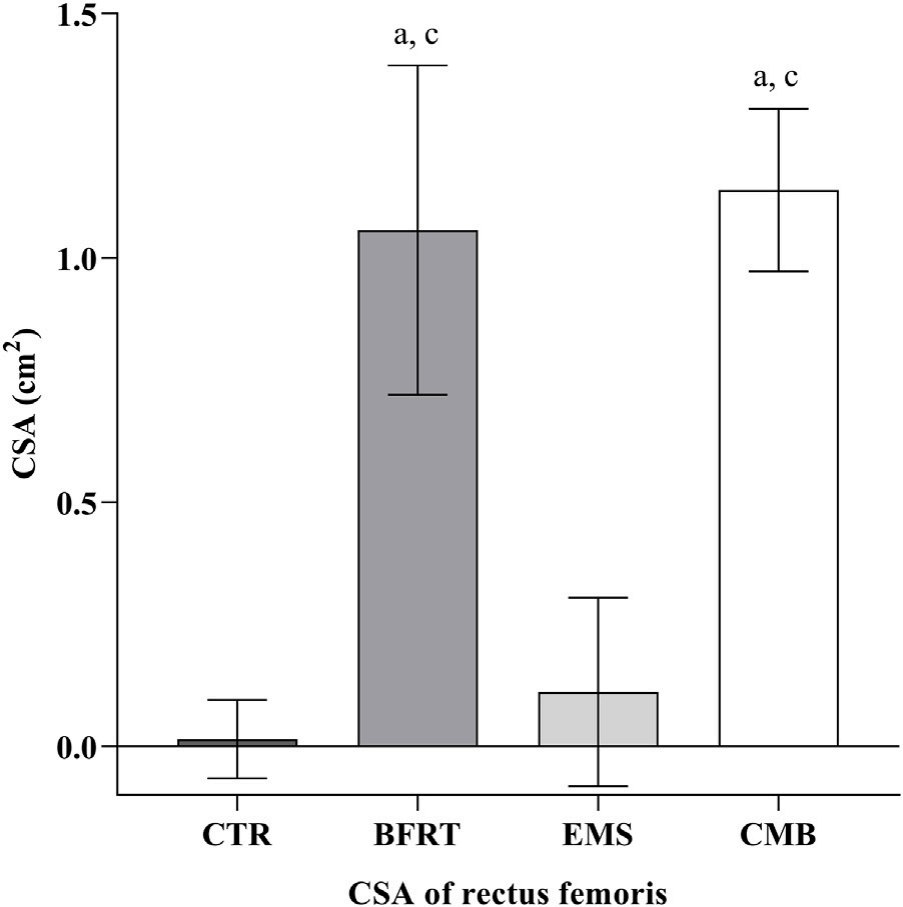
The CSA of rectus femoris in each group. Letters a, b, and c represent comparison with CTR group, BFRT group, and EMS group, respectively. Difference from CTR, ^a^ *p* < 0.05; Difference from BFRT, ^b^ *p* < 0.05; Difference from EMS, ^c^ *p* < 0.05.

Following the 6-week intervention, the changes in CSA, separated by all groups, are shown in Figure 5. There was a greater CSA increase in the CMB group compared to the EMS group (*p* < 0.05) and the CTR group (*p* < 0.05), but was not different from that in the BFRT group (*p* = 0.916). It also confirmed a greater CSA increase in the BFRT group than in the CTR group (*p* < 0.05) and the EMS group (*p* < 0.05). However, there was no significant change between the CTR group and the EMS group (*p* = 0.262).

### 3.3 Muscle activation

**FIGURE 6:**
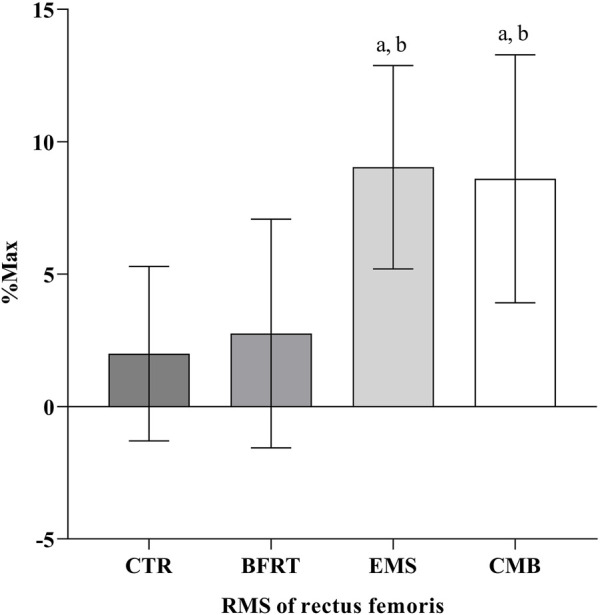
The RMS of rectus femoris in each group. Letters a, b, and c represent comparison with CTR group, BFRT group, and EMS group, respectively. Difference from CTR, ^a^ *p* < 0.05, ^aa^ *p* < 0.01; Difference from BFRT, ^b^ *p* < 0.05; Difference from EMS, ^c^ *p* < 0.05.

Following the 6-week intervention, the changes in RMS, separated by all groups, are shown in Figure 6. There was a greater RMS increase in the CMB group compared to the BFRT group (*p* < 0.05) and the CTR group (*p* < 0.05), but was not different from that in the EMS group (*p* = 0.986). It also confirmed a greater RMS increase in the EMS group than in the CTR group (*p* < 0.05) and the BFRT group (*p* < 0.05). However, there was no significant change between the CTR group and the BFRT group (*p* = 0.934).

### 3.4 Muscle strength

**FIGURE 7:**
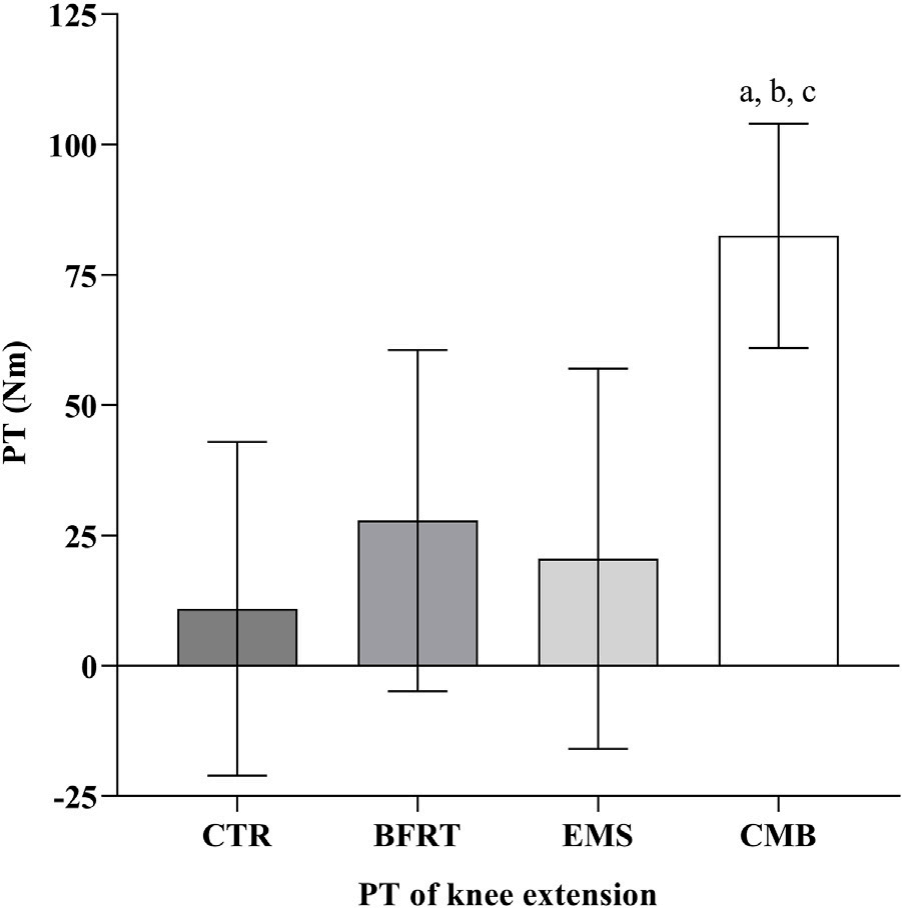
The PT of knee extensor in each group. Letters a, b, and c represent comparison with CTR group, BFRT group, and EMS group, respectively. Difference from CTR, ^a^ *p* < 0.05, ^aa^ *p* < 0.01; Difference from BFRT, ^b^ *p* < 0.05; Difference from EMS, ^c^ *p* < 0.05.

Following the 6-week intervention, the changes in PT, separated by all groups, are shown in Figure 7. There was a greater PT increase in the CMB group compared to the CTR group (*p* < 0.05), the EMS group (*p* < 0.05), and the BFRT group (*p* < 0.05). However, there was no significant change in the BFRT group compared to the CTR group (*p* = 0.321), and the EMS group (*p* = 0.879). And also, there was no significant change in the EMS group compared to the CTR group (*p* = 0.763).

## 4 Discussion

Considering the important role of lower extremity muscle strength in daily activities and sports, as well as a more suitable exercise mode for clinical skeletal muscle rehabilitation patients, elderly individuals with muscular atrophy, and other special populations, this study chose low-intensity (25% 1RM) squat training as the exercise intervention method. The applications of this method could have important implications for sports rehabilitation.

Studies on the process of muscle strength growth have shown that early strength growth (0–10 weeks) in the early phase is due to increased neuromuscular adaptation, while strength growth in the later period (after 10 weeks) is related to muscle hypertrophy (Kraemer et al., 1996). In this study, BFRT combined with low-intensity squat training was used to induce significant muscle hypertrophy after 6 weeks, proving that BFRT combined with low-intensity resistance training seems to produce a muscle hypertrophy effect similar to that of the traditional high-intensity resistance exercise, which is consistent with the results of previous studies. At present, the research on the effect of EMS is mostly focused on muscle strength (de Oliveira et al., 2021; Nishikawa et al., 2021; Rodrigues-Santana et al., 2021), and there are few studies on the effect of muscle volume. In this study, it was found that 6 weeks of EMS combined with low-intensity squat training could promote the volume of RF, but the improvement effect was significantly less than that of the BFRT group and CMB group. This may be related to the intensity of electrical stimulation and the intervention cycle selected in this experiment. To avoid the discomfort caused by electric current to the participants, the minimum threshold value (75 Hz), which can effectively stimulate the increase in muscle strength, was selected in this study. In terms of the combination of BFRT and EMS, Gorgey et al. found that the acute effects of EMS + BFRT may suggest that an increase in flow-mediated dilation may partially contribute to skeletal muscle hypertrophy, and BFRT combined with EMS could be a strategy to increase skeletal muscle size (Gorgey et al., 2016). Natsume et al. found in animal experiments that BFRT combined with EMS can induce muscle hypertrophy more than BFRT alone, resulting in increased protein expression levels related to muscle hypertrophy and accumulation of metabolites, as well as promoting muscle fatigue and increasing gastrocnemius muscle mass (Natsume et al., 2019). The results of this study showed that the combined BFRT and EMS could induce muscle volume changes more than EMS alone, which was consistent with previous studies, but the specific mechanism needs to be further studied.

Increased recruitment of muscle fibers is thought to be one of the underlying neural mechanisms that cause muscle strength to increase (Loenneke et al., 2011). However, our study found that combined BFRT and EMS were significantly better than BFRT alone in promoting muscle activation, and BFRT combined with low-intensity squat training did not seem to produce the same increase in muscle activation as the traditional high-intensity training (Manini and Clark, 2009; Cook et al., 2013). The main factor by which BFRT combined with low-intensity squat training improves muscle strength may be the muscle hypertrophy effect, while neuromuscular adaptation has little effect. Yamanaka et al. showed that compared with low-intensity BFRT (30% 1RM), the training effect of high-intensity training (75% 1RM) and combined training (2 days low-intensity BFRT+1 day high-intensity training) was mainly due to neural adaptation (Yamanaka et al., 2012). Therefore, it was suggested that BFRT can be used in combination with traditional high-intensity training to produce more effective stimulation of muscle volume and neuroadaptation. Previous studies have found that BFRT can cause nerve numbness and other adverse reactions (Cook et al., 2013). Therefore, we believe that the failure of BFRT to enhance neuromuscular adaptability may be related to the limitation of nerve conduction while restricting blood flow. At present, no study has explicitly investigated the effect of BFRT combined with EMS on the electrical activity of muscles. The results of this study found that compared with low-intensity training + BFRT, low-intensity training combined with BFRT + EMS had a better effect on the increase in muscle activation. The combination of BFRT and EMS can simultaneously achieve effective improvement of muscle volume and neuromuscular adaptation. Training combined with EMS can compensate for the weakness of BFRT limiting the increase in muscle fiber recruitment, and low-intensity training is safer than high-intensity training. In addition, studies have shown that after BFRT, the concentration of inorganic phosphate in muscle increases significantly, resulting in more obvious peripheral fatigue (Head et al., 2020). After BFRT, inorganic phosphate in type I and type II muscle fibers was rapidly consumed, and EMS increased muscle fiber recruitment to maintain power output. This may also be a possible mechanism for increased muscle activation in the CMB group.

The role of BFRT in promoting muscle strength has been confirmed by a large number of studies (Shinohara et al., 1998; Takarada et al., 2000). The results confirmed that BFRT combined with low-intensity squat training can improve the muscle strength of participants who exercise irregularly, and it was believed that the main reason for the improvement of the lower limb muscle strength caused by BFRT combined with low-intensity squat training may be related to the significant increase in muscle volume.

The improvement of muscle strength depends not only on the increase in muscle volume but also on the increase in motor unit recruitment. EMS can improve the innervation of the central system and coordinate the agonistic and antagonistic muscles, thus promoting the recruitment of motor units, activating more muscle fibers, and enhancing muscle strength (Kemmler et al., 2018). The mechanism by which EMS promotes muscle strength is closely related to the intensity and frequency of stimulation. Cunha et al. found that 8 weeks of EMS training (70 Hz) can effectively improve volleyball players’ lower limb muscle strength and explosive power (da Cunha et al., 2020). Toth et al. pointed out that EMS (400 µs, 50 Hz) can prevent quadriceps atrophy after ACL surgery and improve the maximum muscle strength of patients (Toth et al., 2020). Pantović et al. showed that EMS and traditional high-intensity resistance strength training had the same training effect in improving muscle strength (Pantović et al., 2015). Our study found that 6 weeks of EMS combined with low-intensity squat training effectively improved lower limb muscle strength, and the intervention effect was similar to that of BFRT combined with low-intensity squat training, which may be related to more significant muscle activation in the EMS group. In terms of the combination of BFRT and EMS, Gorgey et al. found that the combination of BFRT and EMS can effectively improve the wrist strength of patients with incomplete quadriplegia (Gorgey et al., 2016). Natsume et al. showed that the combination of BFRT and EMS can significantly increase the maximum knee extension strength of young men with irregular exercise habits (Natsume et al., 2019). Slysz et al. found that 6 weeks of EMS combined with BFRT training effectively improved the leg lift strength of lower limbs (Slysz and Burr, 2018). The above scholars all believe that the increase in muscle strength is mainly related to muscle hypertrophy. However, the results of this study showed that when BFRT was used, whether or not EMS was used together had a significant influence on muscle strength. Similarly, when using EMS, whether BFRT was combined with EMS also had a significant influence on muscle strength. This suggests that the increase in muscle strength caused by BFRT and EMS is related to neuromuscular adaptation and muscle hypertrophy.

The current study has some limitations. First, the sample of this study was restricted to young, healthy men. Thus, we acknowledge that our findings may not apply to other populations. In future studies, patients with musculoskeletal diseases can be recruited as research participants to further verify the clinical application value. Second, due to a large number of measurements and participants involved in this experiment, all measurements were only collected before and after the intervention. Therefore, future studies can appropriately focus on certain major measurements and exclude individual differences between participants through the measurement at different times.

## 5 Conclusion

The combination of BFRT and EMS for low-intensity squat training can improve the muscle strength of lower limbs by promoting muscle hypertrophy and increasing muscle activation, which makes up for the limitations and deficiencies of each intervention method used alone.

## Data Availability

The raw data supporting the conclusion of this article will be made available by the authors, without undue reservation.
